# Stomatitis Materna

**Published:** 1860-04

**Authors:** L. S. Ellis

**Affiliations:** Chicago, Ill.


ARTICLE 3.

### STOMATITIS MATERNA.

BY L. S. ELLIS, M. D., CHICAGO, ILL.

This disease has, of late, elicited much attention from the medical profession. Its severity and obstinacy have been not a little magnified; its pathology pushed far into the mysterious; its treatment as varied as speculation, and speculation verged near upon absurdity. It has been represented as a new disease, which the oldest practitioners "have never yet met with," and a recent writer "could not find a single reference to this disease in any of the works of Practice, or Special Diseases of Females."

Dr. Dunglison, in his Practice, printed in 1842, vol. 1, p. 31, says: "It is not common with us, but it is said to be very often met with in moist countries, as in Holland, where it reigns at times epidemically, and is a serious affection, attacking adults, and child-bed females especially." Again, on p. 32, "The author has observed some obstinate cases in women who were nursing, the affection appearing to be induced by the constant drain from the mother interfering with nutrition."

In Bell & Stokes' Practice, 1845, I find, "There is yet a kind of sore mouth, which, as far as our present knowledge extends, is seen only in women during lactation." Vol. 1, page 52.

Dr. Wood gives the disease as extended review, as the relative importance of the disease demands.

The medical literature of this malady is more abundant, and in our country, extends back at least thirty years. I have not yet seen any such evidence as to induce me to believe that the disease is of modern date. The great source of modern improvement in medicine consists in a more accurate isolation and discrimination of particular disease. This accurate discrimination of disease alike tends to the advancement of our profession, and to individual success. In many cases what were considered symptoms by old authors, are now exalted into the ranks of distinct diseases. Also, by reference to authors as old as Cullen, we find whole groups of distinct diseases grouped under one head or class, determined by locality. Thus, under the title of Hepatitis, Cullen gives but three pages to diseases of the liver. While thus he makes no mention of such diseases as *fatty degeneration* or *cyrrhosis*, no one will claim that such maladies had no existence in his time. I believe that wherever a like combination of influences exists, there like consequences will follow.

Views of the pathology of this disease are as diverse as the localities of the authors. A recent writer says, "In all these cases I have seen inflammation of the cervix uteri and of the superior portion of the vagina." Another finds the *causa belli* in "miasmata," in "biliary derangement," and in "the influence of gestation and lactation." Another dives deep into that medical arcana, the blood. That inner temple of mysteries holds a wondrous amount of vagaries, whose warrings, if visible, would reveal to us a terrible pandemonium in that "liquid flesh" of ours.

The most positive testimony exists as to the unlimited extent of the prevalence of the disease. Proof is as abundant as of any disease whose history has been so recently written. None will doubt its existence in Illinois. Prof. M. M. Pallen speaks of it in Missouri; Dr. Shanks in Tennessee; Dr. Brandon in Georgia; Dr. Armor in Ohio; Dr. Dunglison in Pennsylvania; Dr. Backus in New York; Dr. Hale in Massachusetts; Dr. Marshall Hall in England; M. Guersent in France and in Holland. Names could be cited by scores, showing that the disease is not confined to malarious districts of our country. It occurs alike on the mountains of New England and New York, as well as on the prairies of Illinois. It exists in England, where Dr. Watson says, "Intermittent fever is known to us only in its group of symptoms." Likewise on the continent its existence with malaria exclusively is without proof. Before we accept this dogma of its malarious relation, we ought certainly to have some *positive* proof of its obeying malarial laws; proof that it occurs in greatest extent and severity at the same seasons; decreases in cities; vanishes with the settlement and cultivation of our country, instead of with the "*early healthy physical developement* of the daughter." On the contrary, I have observed it at all seasons, following not in the channel or seasons of epidemic malaria, but after fortuitous occurrences with those whose idiosyncracies predispose to it.

As before intimated, the *blood* has been obliged to assume the burden and responsibility of this disease. As a scapegoat of physical aberration, it has borne, not only the disease in its obscurity, but its task-masters into still greater mysticism. In this connection I cannot forbear quoting from a recently promulgated theory, especially illuminating:

"Of its dependence upon the depravity of the blood, I have no doubt. I consider the health of this vital fluid dependent upon the harmonious action of all the assimilative organs of the body, and a due supply of *pabulum* of proper blood food.

"If any one of the tissues fail to be nourished by an error of nutrition, I think the elements of that tissue *remain* as abnormal constituents of the blood; and that the elimination of those particular elements by their natural elective power, *alone* can render it normal for the production of the other tissues.

"The developement of *hair* upon the *fœtus* in utero, is in obedience, I think, with *this* general law. We know of no other physiological utility, as it almost always, very soon after birth, gives place to a reproduction. If its elementary constituents are eliminated in the progress of fœtal development, that the *blood*, after this assimilation, might be in its integrity for a second assimilation to nourish another tissue; and the succession of these assimilations maintain not only the proper constituents, but the constant normal catalysis of the *blood*, then we see how necessary are the developement of all the uses *in utero*, as well as after birth. And the development of rudimentary structures in the *one sex* when the requirements of organs are confined to the *other*, as in case of the mammary gland, etc."

To know what the above means, what are its relations to Stomatitis Materna, and how it proves it a blood disease, the inquirer must refer to Trans. Ill. State Med. Society, 1859, p. 43, et. seq."

It is "a blood disease?" If so where is the proof? Do the speculations of writers make any approximation to proof? Does the successful therapeutical management of the disease, even by such theorists themselves, afford any support to their theories? If any knowledge of practical value is to result from theory, it seems it ought, in some manner, to be supported by microscopical investigations, corelative symptoms, and therapeutical applications. Dyspepsia may be accompanied by a "paucity of blood corpuscles." Is it, therefore, a blood disease? Prolonged lactation will favor "impoverishment of blood, impair nervous agency, and derange the secretions." Is it a blood disease? Why not come to the obvious and palpable conclusion, that the stomach is primarily at fault—that the "impoverished blood," the "deranged biliary secretions," the "constipated bowels," the "irritated stomach," the "acid urine and saliva," and the ulcerated tongue, are but symptoms of the central derangement—are but indices of impaired digestion, not in the nursing woman alone, but in the dyspeptic man and child.

In all cases falling under my notice, I have found evidence of impaired function of the stomach. This will be found a leading characteristic of the malady if attention is directed to it. These functional derangements do not follow as a sequence of the stomatitis, but are found to precede it in many cases a long time. Women who have had this disease many times, learn by experience to avoid indigestible substances, especially such as induce acidity of the stomach. I have observed connected with this disease, loss of appetite and taste, acidity of stomach, gastric uneasiness after eating, constipation or diarrhœa, persistent or alternating, biliary derangement &c. Following these as a sequence in long continued cases, comes in a long train of "diminution of blood corpuscles," "anemia," "land scurvy," modification of condition of blood," &c., of various authors.

Rapid decay of the teeth is also apt to occur in this train of symptoms; a circumstance I have always referred to the acid secretion acting on the teeth. Herein is the explanation of so many American women losing their teeth, especially in the first lactation. This seems to me to be as much entitled to a place in the category of blood diseases as Stomatitis Materna. If the above described condition of the stomach continues unabated, the decay of the teeth is almost sure to occur, more sure than the disease under consideration.

I am not alone in this mention of gastric derangement. Dr. Dunglison, in his Practice, vol. 1, p. 31, says: "This form is accompanied by great cephalic, gastric, and general disturbance, and at times the eruption appears to extend to the intestinal canal, giving rise to severe pain in the abdomen, diarrhœa, aud typhoid symptoms, under which the patient may sink."

Dr. J. H. Hollister says: "In a great number of instances the disease has made its appearance as the immediate sequence of constipated bowels, and was relieved almost as soon as the proper correction was made." Vide Trans. Ill. Med. Society, 1859, page 47.

Dr. D. S. Brandon, after enumerating the mouth symptoms, says: "To these may be added, burning in the stomach, with occasional vomiting, constipation, or diarrhœa, more or less obstinate." Southern Med. & Surg. Jour. Jan. 1860, p. 4.

I am now in attendance on Mrs. R., who is seven months in pregnancy. She complains of acidity of stomach, and evidences of gastric derangements, and the urine is so intensely acid as to produce severe inflammation and excoriation of the external parts. She has had Stomatitis Materna during previous lactations, both here and in Central New York. I look for the same sequence after her confinement, unless that condition of the stomach is relieved, but not otherwise. In one of the worst cases I ever saw, Mrs. H., it always accompanied acidity and was readily relieved by tonics and antacids. I could point to case after case presenting the same association of symptoms, readily relieved by remedies addressed to the stomach, so that I am accustomed to direct my attention to that organ, and am not inclined to look upon the disease as so uncontrollable and unpromising as many writers.

Much of our knowledge of the *character* of disease is derived from observance of the *effects* of remedies. An important hint, as to the pathology of this disease, may be obtained by reviewing the various remedies proposed by different writers. Dr. Backus may have been hypothetical in his opinion of this malady, but he certainly indicated a principle in its treatment, the wisdom of which is evidenced by its general adoption. His prescription is as follows:

| | |
|---|---|
| ℞—Carb. Ferri. | grs. xlv. |
| Pulv. Rhei. | |
| Gum Aloes | āā grs. xv. |
| Pulv. Ipecac. | |
| Sapo. Hisp. | āā grs. xij. |

M. Ft., Pill No. 50. "Two of these pills should be taken twice or three times a day or often enough to keep the bowels *very open.*"

Dr. Hale rests the cure chiefly on "tonics, such as lime water and infusion of bark." Dr. Wood says "the most efficient remedies are said to be tonics, antacids and laxatives." Dr. D. S. Brandon, in a paper before referred to, lauds turpentine, combined with castor oil or laudanum, according to the state of the bowels; "say twelve drops three or four times a day, on a little loaf sugar, and in no instance that I know, or have heard of, has it failed."

Dr. Armor proposes the syrup of hypophosphites, combined with Sime's Elixir of Peruvian Bark. It may be surmised that the Elixir has as much to do with the cure as the hypophosphites.

I am accustomed to use the two following prescriptions with uniform success:

℞—Mag. Calc. ʒ i.

Sapo. Hisp. pulv. grs. x.

Camphor "

Sang. Can. " āā grs. v.

Mix. Dose from three to five grains, four times daily.

℞—Cinch. Rub. ℥ ss.

Ferri. Carb. Prace.

Rad. Rhei. āā ʒ ij.

Port Wine Oj.

Ft. Mist. Dose, table-spoonful with each meal.

When there is loss of appetite I direct the tonic to be taken half an hour before eating, and the powder soon after, otherwise both are to be taken soon after eating. In many cases where there is not much constitutional debility, and in the earlier stages I find the first prescription amply sufficient to control the disease.

Many local applications have been proposed; among them nitrate of silver has received its due amount of laudation. I have seen this used repeatedly, often with no benefit, and at most affording but temporary relief. With correct views of the pathology of the disease, such an event might be anticipated. When I hear of a physician using this treatment I am constrained to think he does not know what he is dealing with—that his opinion of the disease does not extend any further than he can see.

Very many writers and practitioners recommend, in severe cases, the *weaning of the child. My experience and observation is such as to lead me to hestitate to recommend any such proceedure.* It may be conservative to the mother's health, but there is a tender offspring, who claims our sympathy and consideration as well as the mother. The chances of rearing children throughout our land are few enough at best—small, indeed, in our cities—and fearful to the child that is weaned in the first weeks of its existence. A recommendation to wean the child in such cases in our city, is but a warning to prepare its shroud. I scarcely know an instance in this city where a child has survived the first two or three years of its perils, when weaned as a cure of stomatitis. I have seen mother's turn away from such counsel, and avow their resolve to suffer on rather than expose their offspring to such fearful odds. There may be, and indeed are, severe cases when this proceedure seems imperative. I speak only against the indiscriminate recommendation of some practitioners, believing that, with proper treatment, a vast majority of mothers will survive and preserve their offspring.

With the above digression I return to the consideration of the pathology of the disease.

*Post mortem* examinations of the disease are not abundant. A few have been made. I take from a valuable contribution to the literature of this disease, the following appearances, as described by Dr. McLean:

"The stomach was almost completely denuded of its mucous coat, with numerous patches of ulceration extending deep into its muscular tissue. A small patch around the pyloric orifice of the stomach, was the only healthy portion." Trans. Ind. Med. Society, 1856.

Many writers have asserted positively that the disease occurs only in females, chiefly during gestation and lactation. Others, on the other hand, declare as assuredly that it occurs also in men, boys, and girls. I can only add to the strength of the latter position. I am certain I have seen it in that class of persons with characteristics as prominent and unmistakable as in nursing women. Its only difference seemed to consist in less persistency, owing to a more ready removal of the causes. I had abundant opportunity to examine a case of the kind occurring to a gentleman about thirty years of age. In this case the stomatitis occurred several times, immediately after eating blackberries preserved in tin cans a long time. Each attack was ushered in by the scalding sensation in the mouth, so intense as to cause severe suffering for several days, by vesicles with inflamed bases, by corrugation of the mucous membrane on the side of the tongue, slight swelling and ulceration of the mucous membrane. In this instance the primary effect was obviously on the stomach. The stomatitis not following immediately, the cause was not discovered till after eating several times.

It may be true that we do not meet with it in so severe a degree as in nursing women. Such might be anticipated. All know that pregnancy and lactation exert a powerful and modifying influence on the stomach, both in health and disease. But I think that here the nervous system is more the medium for communicating sympathetic influence than the blood.

"Its attacks are not confined to any particular constitution or temperament, but are at times made on the most robust, who always enjoyed good health." Bell & Stokes, vol. 1, page 53.

This accords with my experience and observation, but is hardly consistent with the exclusive ancemic hypothesis of some writers.

It does not come within the scope of my object to give a detailed account of the local, mouth symptoms of this malady. They have been already sufficiently indicated in the foregoing. My object has been to indicate a few points in the history and character of this malady. If the position assumed is correct, the mouth symptoms become comparatively unimportant to the practitioner. His remedies must be directed to the stomach. My aim has been to substantiate the following positions.

*First.* That the history of the disease extends too far back to entitle it to the claim of a new disease.

*Second.* That so far as known it occurs in all localities and is not limited to malarial districts.

*Third.* That the primary seat of the malady is in the stomach, modified in the female by gestation and lactation, and followed by constitutional aberration as *sequences.*

This is sustained, 1st, by correlative symptoms; 2d, by therapeutical applications; 3d, by post mortem examinations.

*Fourth.* That nursing women are the chief sufferers, but that it occurs in other classes of persons.

